# Supplementary material for: Novel Cyclic Homogeneous Oscillation Detection Method for High Accuracy and Specific Characterization of Neural Dynamics
Source: bioRxiv. 2024 Mar 23:2023.10.04.560843. Preprint. [Version 2] doi: 10.1101/2023.10.04.560843 (PMC10983872; doi:10.1101/2023.10.04.560843)
Supplement: Supplement 1 [file NIHPP2023.10.04.560843v2-supplement-1.pdf]

bioRxiv preprint doi: https://doi.org/10.1101/2023.10.04.560843; this version posted March 23, 2024. The copyright holder for this preprint (which was not certified by peer review) is the author/funder, who has granted bioRxiv a license to display the preprint in perpetuity. It is made available under aCC-BY 4.0 International license.

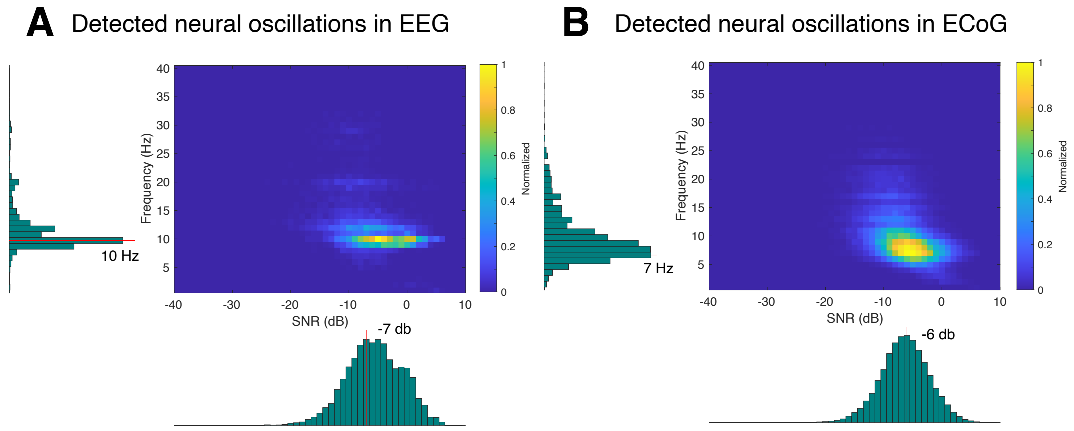

Figure 4—figure supplement 1. SNR Histograms of EEG (A) and ECoG (B).

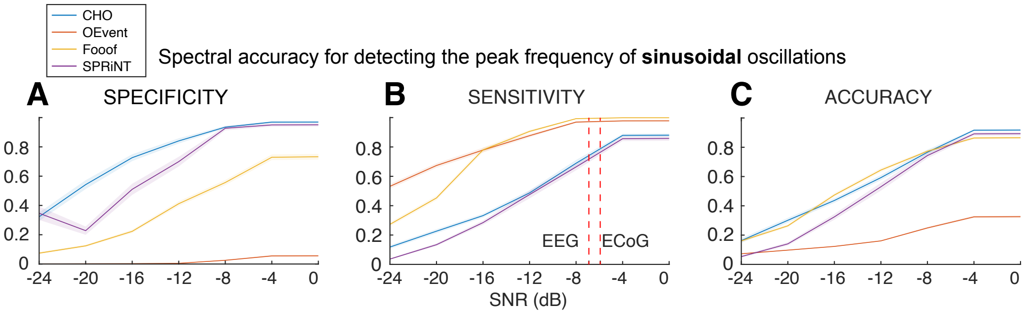

Figure 4—figure supplement 2. Synthetic sinusoidal oscillations.

bioRxiv preprint doi: https://doi.org/10.1101/2023.10.04.560843; this version posted March 23, 2024. The copyright holder for this preprint (which was not certified by peer review) is the author/funder, who has granted bioRxiv a license to display the preprint in perpetuity. It is made available under aCC-BY 4.0 International license.

927

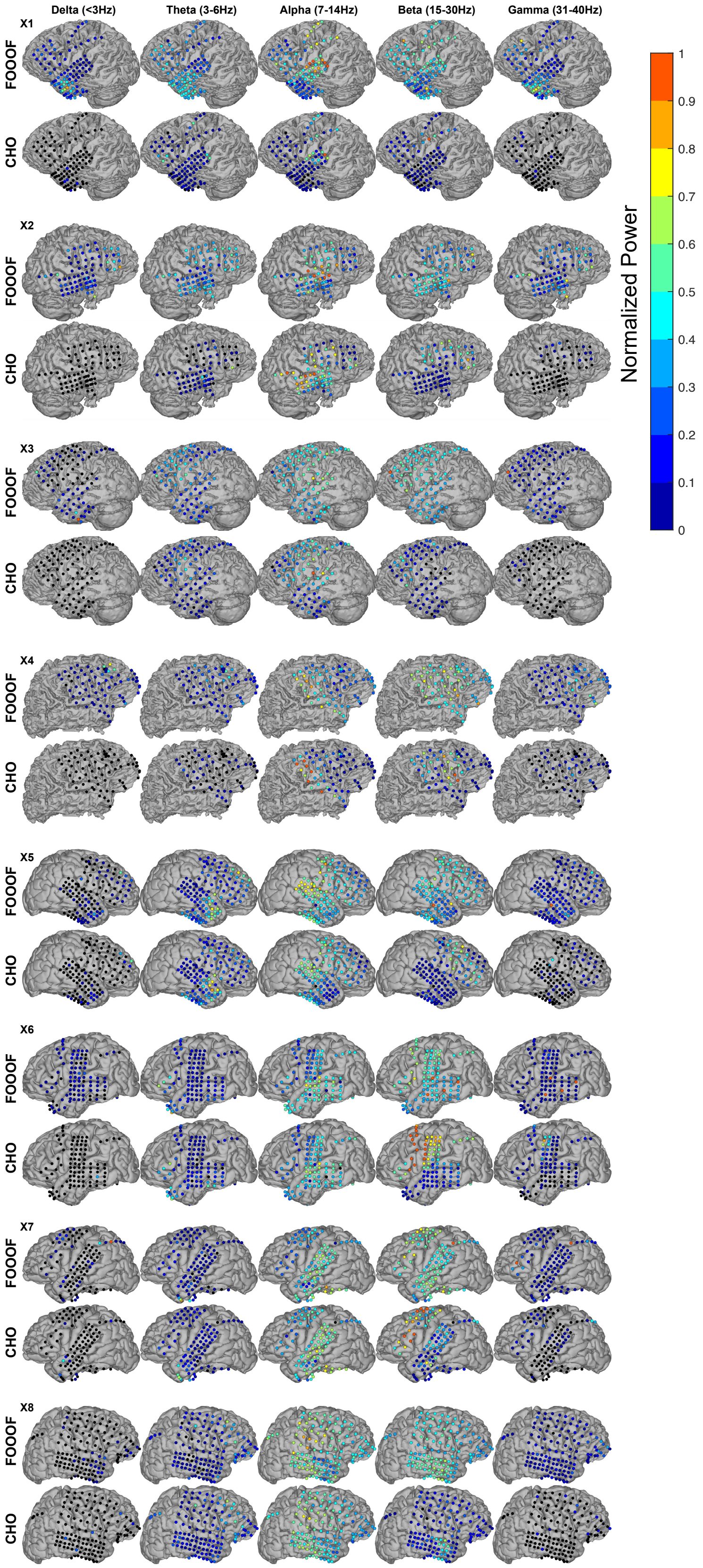

Figure 5—figure supplement 1. ECoG results using FOOOF and CHO for all subjects.

bioRxiv preprint doi: https://doi.org/10.1101/2023.10.04.560843; this version posted March 23, 2024. The copyright holder for this preprint (which was not certified by peer review) is the author/funder, who has granted bioRxiv a license to display the preprint in perpetuity. It is made available under aCC-BY 4.0 International license.

928

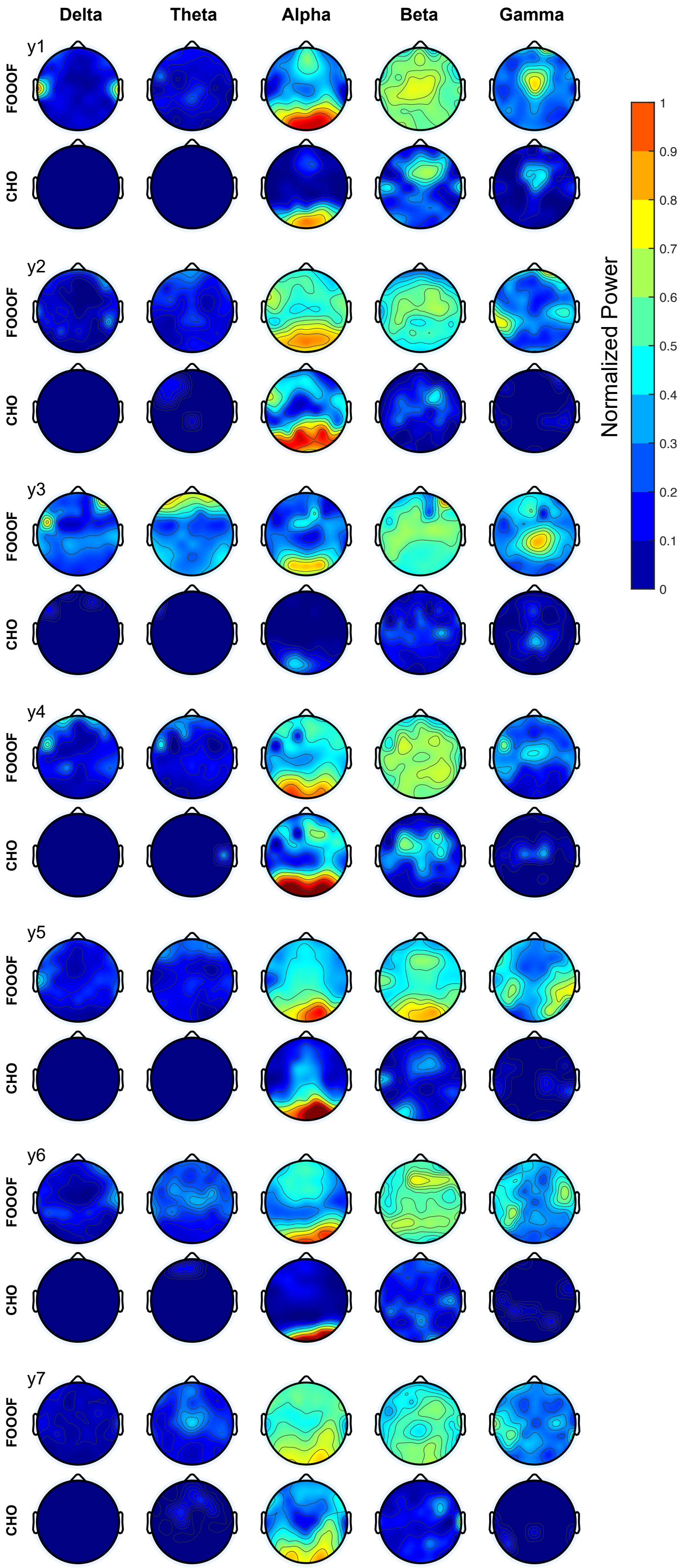

Figure 6—figure supplement 1. Results from seven EEG subjects using the FOOOF and CHO methods.

bioRxiv preprint doi: https://doi.org/10.1101/2023.10.04.560843; this version posted March 23, 2024. The copyright holder for this preprint (which was not certified by peer review) is the author/funder, who has granted bioRxiv a license to display the preprint in perpetuity. It is made available under aCC-BY 4.0 International license.

929

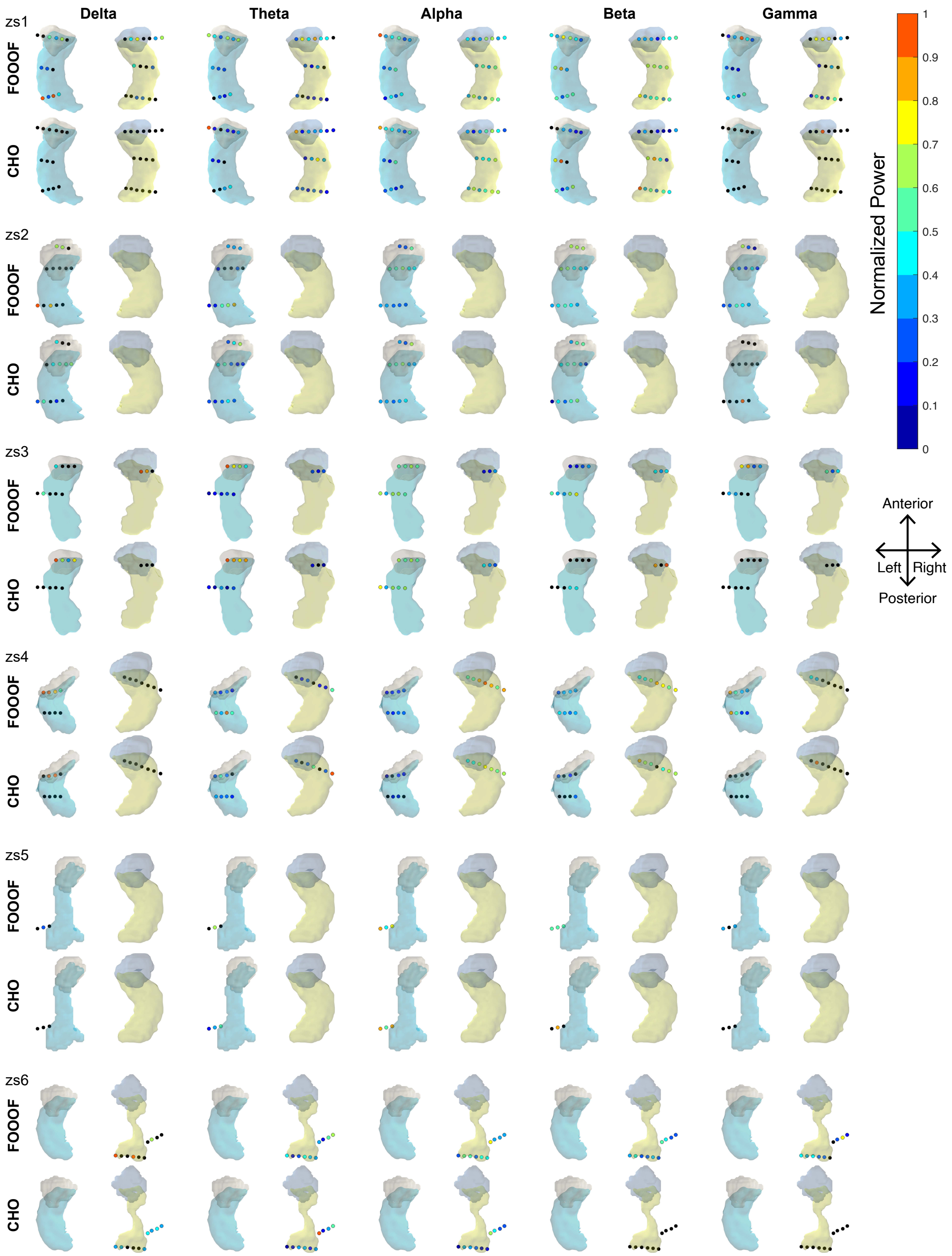

Figure 8—figure supplement 1. All results from six SEEG subjects using the FOOOF and CHO methods.
